# HMGB1/RAGE Signaling and Pro-Inflammatory Cytokine Responses in Non-HIV Adults with Active Pulmonary Tuberculosis

**DOI:** 10.1371/journal.pone.0159132

**Published:** 2016-07-19

**Authors:** Grace Lui, Chun Kwok Wong, Margaret Ip, Yi Jun Chu, Irene M. H. Yung, Catherine S. K. Cheung, Lin Zheng, Judy S. Y. Lam, Ka Tak Wong, Winnie W. Y. Sin, Kin Wing Choi, Nelson Lee

**Affiliations:** 1 Department of Medicine and Therapeutics, Prince of Wales Hospital, Hong Kong SAR, China; 2 Department of Chemical Pathology, The Chinese University of Hong Kong, Hong Kong SAR, China; 3 Department of Microbiology, The Chinese University of Hong Kong, Hong Kong SAR, China; 4 Department of Imaging and Interventional Radiology, Prince of Wales Hospital, Hong Kong SAR, China; 5 Department of Medicine, Alice Ho Miu Ling Nethersole Hospital, Hong Kong SAR, China; 6 Stanley Ho Center for Emerging Infectious Diseases, The Chinese University of Hong Kong, Hong Kong SAR, China; Karolinska Institutet, SWEDEN

## Abstract

**Background:**

We aimed to study the pathogenic roles of High-Mobility Group Box 1 (HMGB1) / Receptor-for-Advanced-Glycation-End-products (RAGE) signaling and pro-inflammatory cytokines in patients with active pulmonary tuberculosis (PTB).

**Methods:**

A prospective study was conducted among non-HIV adults newly-diagnosed with active PTB at two acute-care hospitals (n = 80); age-and-sex matched asymptomatic individuals (tested for latent TB) were used for comparison (n = 45). Plasma concentrations of 8 cytokines/chemokines, HMGB1, soluble-RAGE, and transmembrane-RAGE expressed on monocytes/dendritic cells, were measured. Gene expression (mRNA) of HMGB1, RAGE, and inflammasome-NALP3 was quantified. Patients’ PBMCs were stimulated with recombinant-HMGB1 and MTB-antigen (lipoarabinomannan) for cytokine induction e*x vivo*.

**Results:**

In active PTB, plasma IL-8/CXCL8 [median(IQR), 6.0(3.6–15.1) *vs* 3.6(3.6–3.6) pg/ml, P<0.001] and IL-6 were elevated, which significantly correlated with mycobacterial load, extent of lung consolidation (***r***_***s***_ +0.509, P<0.001), severity-score (***r***_***s***_ +0.317, P = 0.004), and fever and hospitalization durations (***r***_***s***_ +0.407, P<0.001). IL-18 and sTNFR1 also increased. Plasma IL-8/CXCL8 (adjusted OR 1.12, 95%CI 1.02–1.23 per unit increase, P = 0.021) and HMGB1 (adjusted OR 1.42 per unit increase, 95%CI 1.08–1.87, P = 0.012) concentrations were independent predictors for respiratory failure, as well as for ICU admission/death. Gene expression of HMGB1, RAGE, and inflammasome-NALP3 were upregulated (1.2−2.8 fold). Transmembrane-RAGE was increased, whereas the decoy soluble-RAGE was significantly depleted. RAGE and HMGB1 gene expressions positively correlated with cytokine levels (IL-8/CXCL8, IL-6, sTNFR1) and clinico-/radiographical severity (e.g. extent of consolidation *r*_s_ +0.240, P = 0.034). *Ex vivo*, recombinant-HMGB1 potentiated cytokine release (e.g. TNF-α) when combined with lipoarabinomannan.

**Conclusion:**

In patients with active PTB, HMGB1/RAGE signaling and pro-inflammatory cytokines may play important roles in pathogenesis and disease manifestations. Our clinico-immunological data can provide basis for the development of new strategies for disease monitoring, management and control.

## Introduction

The global burden of tuberculosis (TB) is enormous, and the disease continues to be a major cause of mortality worldwide [1,2]. It is estimated that over 2 billion people are infected; and in 2014 alone, 9.6 million people developed active disease, resulting in 1.5 million deaths [1]. Even in middle-to-high income countries where anti-TB treatment is more accessible, mortality of patients hospitalized for TB remains high (>5–10%) [3,4]. It is suggested that excessive inflammation contributes to disease severity and complications in active TB; however the immunopathogenic processes involved have not been fully elucidated [5]. Recent studies indicate that in addition to cell-mediated immunity, innate immune responses and the pro-inflammatory cytokines play significant roles in mycobacterial control, while exacerbating tissue damage [5,6]. Better understanding of these mechanisms in clinical infections may lead to development of novel strategies for TB disease management and control [6–9].

High mobility group box 1 (HMGB1), a nuclear DNA-binding protein, is one of the key danger-associated molecular patterns (DAMPs) that activate the innate immune system [8]. It is released by necrotic cells and monocytes/macrophages in response to cytokine and bacterial antigen stimulation [8,10,11], and shown to be inflammasome (caspase-1-activating, multi-protein complexes containing a sensory protein, e.g. NALP3) dependent in bacterial infections [12–14]. HMGB1 then interacts with other molecules (e.g. bacterial LPS), and bind to receptors including the Receptor for Advanced Glycation End products (RAGE, exists in both cellular ‘transmembrane’ form, and a ‘soluble’, decoy receptor form) and the Toll-like Receptors (e.g. TLR2), to activate a multitude of pro-inflammatory genes [8,10,11,12,15]. In addition to liberation of inflammatory cytokines (e.g. TNF-α), HMGB1 will activate immune cell functions and induce their maturation (e.g. monocytes, myeloid/plasmacytoid dendritic cells) [8,10,11]. The strong pro-inflammatory role of HMGB1 has been shown in autoimmune diseases, trauma, sepsis, and bacterial pneumonia [8,10,14,16,17]. Data on TB however, is scarce. Limited *in vitro* and animal studies have shown that *M*. *tuberculosis* (MTB) [18] and *M*. *bovis* BCG [19] can effectively induce secretion of HMGB1, leading to cytokine hyperactivation and lung tissue damage. Recently, increased HMGB1 in serum of MTB-infected patients is reported but its significance is uncertain [20]. In the current study, we hypothesized that HMGB1/RAGE signaling and the pro-inflammatory cytokine responses play significant roles in pathogenesis and disease manifestations in patients with active pulmonary tuberculosis (PTB). Patients presented with active PTB were compared with latent TB and non-infected asymptomatic individuals. Relationships between immunological variables and disease severity were analyzed. The cellular mechanisms involved in the signaling processes were studied.

## Materials and Methods

### Study design and subjects

A prospective study was conducted on adults hospitalized for laboratory-confirmed pulmonary tuberculosis (PTB) over a period of 24 months (November 2011–November 2013) at two acute, general public hospitals operating under the Hospital Authority of Hong Kong, which serve an urban population of >1.5 million [4,21]. Hong Kong is classified as an area of intermediate TB burden, with an annual incidence about 80–100 per 100,000 population; multidrug resistance and HIV co-infection are rare [1,4,22]. Adults aged 18 years or above, presented with and diagnosed active PTB during the current episode of hospitalization were consecutively recruited. Active PTB was defined as the presence of symptoms (respiratory and systemic) and radiological findings indicative of active tuberculous infection, and confirmed by positive acid fast bacilli (AFB) staining, mycobacterial culture, PCR, histological findings, or a combination of these tests, as previously described [4,21,22]. Patients with recent trauma, HIV/AIDS (routinely tested in TB cases in Hong Kong), active malignancy, autoimmune diseases and long-term immunosuppressant use were excluded because of possible induction/suppression of HMGB1 [10,11]. Age- and sex-matched asymptomatic adults were recruited from the general medical, out-patient clinics for comparison; latent TB infection was diagnosed by an interferon-gamma release assay (IGRA, see below). Exclusion criteria were symptoms suggestive of any form of active infection, history or radiographic evidence of TB, and underlying compromising conditions aforementioned. In Hong Kong, prevalence of latent TB is high among the general population, as shown previously [21]. Informed written consent was obtained for every enrolled subject; ethics approval for the study was obtained from the Institutional Review Board of the Hospital Authority of Hong Kong and The Chinese University of Hong Kong.

### Study procedures and definitions

Active PTB cases were identified by the clinical/laboratory research team once the diagnosis was established; clinical data, peripheral blood and sputum samples were collected immediately after recruitment. A standardized research tool was used to collect clinical data [4,21], including demographics, co-morbidities (as defined in the Charlson comorbidity index)[23]; 11 clinical variables (fever, cough, hemoptysis, dyspnea, chest pain, night sweating, pallor, tachycardia, lung auscultation abnormalities, body weight and height, mid-upper arm circumference) for the calculation of ‘TB severity score’[24]; concomitant extra-pulmonary involvement; hypoxemia requiring supplemental oxygen support; durations of fever and hospitalization [4,25,26,27]; and outcomes of ICU admission and death that occurred within the same episode of hospitalization [4]. All plain frontal chest radiographs performed at admission were reviewed by an independent radiologist (LJSY) blinded to clinical information. Radiographic changes (consolidation, cavitation, miliary nodules, effusion) and the percentage of lung parenchyma affected was recorded, using methods previously described [21,28,29].

A total of 25 ml of peripheral blood (EDTA) was collected from each subject for cytokine/chemokine, HMGB-1, RAGE, inflammasome, gene expression and *ex-vivo* stimulation studies (see below). Expectorated sputum samples provided before anti-TB treatment were subjected to mycobacterial culture in liquid medium (MGIT, Bactec culture system); the ‘time-to-culture positivity’ (defined as the number of days after inoculation until a positive signal was obtained, shown to be inversely proportional to log_10_ CFU of inoculum) was recorded to provide an estimate on the mycobacterial load [30]. Semi-quantitative AFB smear results (WHO grading) were also recorded. For the asymptomatic individuals (tested HIV-negative), an IGRA (QuantiFERON-TB Gold In-Tube, Cellestis, Australia) was used to diagnose latent TB infection as described [21].

### Assays of plasma HMGB1, soluble RAGE, and cytokines/chemokines concentrations

Plasma concentrations of interleukin(IL)-1β, IL-6, IL-8/CXCL8, IL-10, IL-12p70, and tumour necrosis factor(TNF)-α were assayed using cytometric bead array (CBA) reagents (BD Pharmingen Corp., San Diego, CA, USA) with four-color FACSCalibur flow-cytometer (BD Biosciences Corp, San Jose, CA, USA) as described [25–27]. Plasma concentrations of HMGB1, soluble RAGE (soluble receptor of HMGB1), IL-18, and sTNFR-1 (soluble tumour necrosis factor receptor-1, which indirectly indicating TNF-α release) were measured using enzyme-linked immunosorbent assays (Shino-Test, Kanagawa, Japan; R&D systems, Minneapolis, MN, USA; and MBL International Corp., Des Plaines, IL, USA) (S1 File). These cytokines/chemokines were selected based on their reported involvement in the pro-inflammatory, innate response in active PTB (the ‘adaptive’ Th1/Th2 cytokines e.g. IFN-γ, IP-10/CXCL-10, IL-4, will be studied separately) [5,6,31].

### Flow cytometric assay of transmembrane RAGE on monocytes and dendritic cells surface

Expression of transmembrane RAGE on surface of peripheral blood monocytes (CD14+), myeloid dendritic cells/mDC (CD16-CD14-CD85k+CD33+) and plasmacytoid dendritic cells/pDC (CD16-CD14-CD85k+CD33-) was assessed by flow cytometric analysis using established methods (S1 File) [27]. Unconjugated polyclonal rabbit anti-human RAGE antibody for flow analysis (Millipore Co., MA, USA), and PE-conjugated goat anti-rabbit IgG (H+L) secondary antibody (Invitrogen Corp., CA, USA) were used for the detection of RAGE. The CD14+ gated population was set to identify the monocyte population. For the analysis of DC, FITC-conjugated CD14 and CD16, PC7-conjugated immunoglobulin-like transcript (ILT)-3/CD85k/CD1c/BDCA2 (Beckman Coulter Inc, CA, USA), and APC-conjugated CD33 (BD Biosciences, San Jose, CA, USA) were used. The DC population was identified as CD14 and CD16 double negative and ILT3 positive population; mDCs and pDCs were differentiated by high and low CD33 expression, respectively. Monocytes (10,000 viable cells) and DC populations (200,000 viable cells) were gated and analyzed with 4-color FACSCalibur flow cytometer (BD Biosciences Corp, San Jose, CA, USA). All results were expressed in Mean Fluorescence Intensity (MFI).

### Real-time quantitative PCR assay of mRNA expression of HMGB1, RAGE, and NALP3 genes

Total RNA of PBMC was extracted, and the mRNA expression of HMGB1, RAGE, and the inflammasome-related NALP3 genes was measured by real-time RT-PCR assay using the Applied Biosystems 48-well StepOne^™^ Real Time PCR System (Applied Biosystems Inc., CA, USA) (S1 File). The relative gene expression of HMGB1, RAGE, and NALP3 was calculated by comparing with the housekeeping GAPDH gene, and expressed as Relative Quantitation (RQ) values [26,27]. In addition, assay of Caspase-1 (inflammasome-related) activity in PBMC was performed using the Caspase-1 colorimetric assay kit (Millipore Corp., MA, USA).

### Ex vivo stimulation studies with recombinant-HMGB1 and Lipoarabinomannan (LAM)

Human PBMC were isolated using a density gradient centrifugation. PBMC (5 × 10^6^/well) were distributed into 96-well plates, and incubated at 37°C in 1 ml of RPMI-1640 medium, supplemented with 10% (v/v) fetal calf serum (FCS) for 24 hours, with or without the following ligands: (1) recombinant-HMGB1; (2) lipoarabinomannans (LAM, a MTB cell wall related glycolipid, a TLR2 ligand); (3) a combination of recombinant-HMGB1 and LAM (for any additive effect); and (4) oligodeoxynucleotide (CpG-ODNs, a TLR-9 ligand; for comparison), to assess for their effects on cytokine stimulation (S1 File). Concentrations of cytokines/chemokines released into the culture supernatant were quantitated by CBA or ELISA as described above. The fold-change in cytokine release with/without ligand stimulation was calculated for individual cytokine (e.g. TNF-α concentration after incubation with recombinant-HMGB1 / with medium alone). A value of >1.0 was considered as a positive response to ligand stimulation [25,27].

### Statistical analyses

Active PTB patients were compared with IGRA-positive and IGRA-negative asymptomatic individuals (ie, with or without latent TB infection) [21]. Sample size calculation was based on our pilot study data (2:1 ratio; PS software, version 3.0.34). Immunological variables were expressed in median (IQR, interquartile range) unless otherwise specified, and analyzed using non-parametric (Mann-Whitney U) tests. Significant variables were further examined for their correlations with parameters indicating mycobacterial load and clinical severity, using Spearman’s rank correlation coefficient (*r*_s_). Moreover, associations with respiratory failure (defined as hypoxemia requiring supplemental oxygen support), and ICU admission/death were examined [25,26]. Variables with P-values <0.10 in univariate analyses were entered into logistic regression models as covariates, together with potential confounders of age, gender, comorbidity and TB severity score to identify independent associations. Adjusted Odds Ratios (OR) and the 95% confidence intervals (CI) were reported. All probabilities were 2-tailed, and a P value of <0.05 was considered to indicate statistical significance. We also calculated Bonferroni-adjusted P value thresholds for the multiple comparisons performed in Tables 1, 2 and 3 for reference purpose. Statistical analysis was performed using the PASW Statistics software, version 17.0.

**Table 1:** Plasma concentrations of pro-inflammatory cytokines/chemokines, HMGB1, and RAGE in patients with active PTB, compared with IGRA-positive and IGRA-negative asymptomatic individuals.

	Active PTB cases(n = 80) ^1^	IGRA-positive, asymptomatic(n = 17) ^2^	IGRA-negative, asymptomatic(n = 27) ^3^	IGRA-positive/negative,asymptomatic(n = 45) ^4^	P-value^[1 vs 2]^	P-value ^[1 vs 3]^	P-value ^[1 vs 4]^
**IL-1β (pg/mL)**	7.2 (7.2–7.2)	7.2 (7.2–7.2)	7.2 (7.2–7.2)	7.2 (7.2–7.2)	0.217	0.561	0.670
**IL-6 (pg/mL)**	9.4 (6.0–20.5)	3.4 (2.6–4.5)	3.1 (2.6–4.2)	3.2 (2.6–4.3)	<0.001**	<0.001**	<0.001**
**IL-8/CXCL8 (pg/mL)**	6.0 (3.6–15.1)	3.6 (3.6–4.8)	3.6 (3.6–3.6)	3.6 (3.6–3.6)	0.003*	<0.001**	<0.001**
**IL-10 (pg/mL)**	3.3 (3.3–3.3)	3.3 (3.3–3.3)	3.3 (3.3–3.3)	3.3 (3.3–3.3)	0.246	0.145	0.061
**IL-12p70 (pg/mL)**	1.9 (1.9–2.4)	2.1 (1.9–2.6)	1.9 (1.9–2.2)	2.0 (1.9–2.3)	0.320	0.792	0.722
**IL-18 (pg/mL)**	1559.9 (1273.2–1906.0)	1514.1 (1117.1–1734.8)	1103.4 (984.1–1622.8)	1161.9 (1004.1–1661.7)	0.155	<0.001**	<0.001**
**TNF-α (pg/mL)**	7.0 (5.8–8.0)	6.0 (3.8–8.0)	7.7 (6.0–9.7)	6.6 (4.4–8.9)	0.072	0.136	0.924
**sTNFR-1 (pg/mL)**	1556.1 (1064.6–2425.1)	1155.3 (1033.7–1512.8)	1023.4 (828.5–1422.7)	1116.7 (886.4–1440.4)	0.030*	0.001**	<0.001**
**HMGB1 (ng/mL)**	3.1 (1.7–4.5)	2.8 (1.0–4.7)	2.0 (1.3–5.0)	2.1 (1.3–4.7)	0.690	0.307	0.386
**soluble RAGE (pg/mL)**	446.4 (220.9–701.8)	807.7 (614.8–1295.1)	797.8 (562.6–971.4)	797.8 (569.3–1017.1)	<0.001**	<0.001**	<0.001**

**Note**: All results are described as median (IQR) unless otherwise specified. One asymptomatic individual had indeterminate IGRA result. All 80 active PTB cases were prospectively and consecutively recruited (refusal, n = 21); 5 patients were excluded based on study criteria or subsequent culture results showed non-tuberculous mycobacteria. Duration of drug exposure was <48 hours (IQR 24–72 hours). Detection limits for IL-1β, IL-6, IL-8/CXCL8, IL-10, IL-12, IL-18, TNF-α, sTNFR-1, and soluble RAGE are 7.2, 2.5, 3.6, 3.3, 1.9, 9.0, 3.7, 0.4, and 1.2 pg/mL respectively (HMGB1, 1.0 ng/mL). Mann-Whitney U test, ** P ≤0.001, * P <0.05; a Bonferroni-corrected P value significance threshold (P ≤0.003) is calculated for reference.

**Table 2:** Expressions of RAGE, HMGB1 and inflammasome in active PTB patients, compared with IGRA-positive and IGRA-negative asymptomatic individuals.

	Active PTB cases(n = 80) ^1^	IGRA-positive, asymptomatic(n = 17) ^2^	IGRA-negative, asymptomatic(n = 27) ^3^	IGRA-positive/negative, asymptomatic (n = 45) ^4^	P-value ^[1 vs 2]^	P-value ^[1 vs 3]^	P-value ^[1 vs 4]^
**Monocytes, RAGE (MFI)**	173.7 (97.7–259.2)	154.2 (92.6–185.9)	137.8 (81.2–197.9)	140.2 (83.2–193.6)	0.271	0.070	0.077
**Total DC, RAGE (MFI)**	100.0 (62.1–192.2)	102.4 (65.5–129.1)	68.9 (53.3–109.4)	84.2 (58.1–126.5)	0.740	0.049*	0.140
**mDC, RAGE (MFI)**	138.0 (25.7–331.9)	131.8 (10.0–312.0)	133.9 (68.2–248.9)	114.0 (10.0–262.7)	0.415	0.955	0.333
**pDC, RAGE (MFI)**	112.8 (10.0–215.7)	10.0 (10.0–10.0)	20.7 (10.0–83.8)	10.0 (10.0–118.5)	0.254	0.240	0.247
**HMGB1 (RQ)**	1.2 (0.6–3.0)	1.0 (0.2–1.4)	1.0 (0.1–1.6)	1.0 (0.1–1.5)	0.058	0.109	0.016*
**RAGE (RQ)**	1.4 (0.6–3.4)	0.4 (0.1–0.7)	0.5 (0.2–1.1)	0.5 (0.1–1.0)	<0.001**	0.001**	<0.001**
**NALP3 (RQ)**	2.1 (1.3–3.9)	1.1 (0.6–1.5)	2.0 (0.9–3.7)	1.4 (0.7–2.9)	<0.001**	0.342	0.006*
**Caspase-1 (μg/mL)**	21.7 (9.3–42.8)	5.4 (2.0–10.2)	6.9 (1.7–24.2)	6.9 (1.7–16.4)	<0.001**	0.012*	<0.001**

**Note**: MFI, mean fluorescence intensity; RQ, relative quantitation of gene expression (mRNA/GAPDH). ** P ≤0.001, * P <0.05; Bonferroni-corrected P value significance threshold (P ≤0.003) is calculated for reference. Plasma IL-6, IL-8/CXCL8 and sTNFR1 concentrations positively correlated with RQ of RAGE (*r*_s_ 0.214, P = 0.018; *r*_s_ 0.280, P = 0.002; *r*_s_ 0.273, P = 0.002 respectively).

**Table 3:** Correlations between plasma cytokine levels, and clinical and bacteriological variables in patients with active PTB (n = 80).

	Consolidation on CXR, %		Duration of fever, day		Duration of hospitalization, day		TB severity score ^1^		Semi-quantitative AFB smear ^2^		Time-to-culture positivity ^3^	
	*r*_*s*_	P-value	*r*_*s*_	P-value	*r*_*s*_	P-value	*r*_*s*_	P-value	*r*_*s*_	P-value	*r*_*s*_	P-value
**IL-6 (pg/mL)**	0.373	0.001**	0.272	0.015*	0.359	0.001**	0.207	0.065	0.264	0.024*	-0.445	0.007**
**IL-8/CXCL8 (pg/mL)**	0.509	<0.001**	0.137	0.230	0.407	<0.001**	0.317	0.004**	0.247	0.035*	-0.335	0.046*
**IL-18 (pg/mL)**	0.024	0.834	-0.043	0.704	0.236	0.041*	-0.027	0.812	0.220	0.061	0.208	0.222
**sTNFR1 (pg/mL)**	-0.088	0.442	0.029	0.799	0.018	0.878	0.073	0.524	-0.058	0.627	-0.101	0.564

**Note: *r***_***s***_ = Spearman’s rank correlation coefficient; * P <0.05, **P <0.01; a Bonferroni-corrected P value significance threshold (P ≤0.01) is calculated for reference.

^**1**^ TB severity score, constructed based on 13 symptoms and clinical signs [24];

^**2**^ semi-quantitative AFB smear results (WHO grading 0, 1+, 2+, 3+);

^**3**^ time-to-positivity in the liquid-medium culture system (shorter time to positivity indicated a higher mycobacterial load; data available in 36 cases)[30]. Correlations between percentage consolidation on CXR, and RQ of HMGB1, RAGE and NALP3 were *r*_s_ 0.204 (P = 0.074), *r*_s_ 0.240 (P = 0.034), and *r*_s_ 0.278 (P = 0.014) respectively. Cavitatory *vs* non-cavitatory disease: IL-8, median(IQR), 8.5(4.3–15.1) vs 5.2(3.6–14.8), P = 0.159; IL-6, 11.6(7.4–24.8) vs 8.8(4.9–20.4), P = 0.099. There was a trend to show RQ of HMGB1 correlated with fever duration (*r*_s_ 0.222, P = 0.053). Higher plasma IL-18 level was found in patients with extra-pulmonary manifestations: median (IQR), 2713.8 (1551.5–3555.8) *vs* 1488.6 (1268.3–1890.1) pg/mL, P = 0.019.

## Results

### Description of patients

Altogether, 80 patients with active PTB and 45 asymptomatic adults were recruited for study. The median (IQR) age of PTB patients was 52 (39–69) years; 45% had underlying comorbidities, and 28% were current smokers. The median duration of symptom prior to presentation was 4 (IQR 2–13) weeks. About 73% of cases were AFB smear-positive in one or more clinical samples; all mycobacterium isolates obtained were tested susceptible to first-line agents. In 6%, concomitant extra-pulmonary manifestations were documented (laryngitis 3, peritonitis 1, osteomyelitis 1). About 61%, 26% and 13% of patients had severity scores classified as class I (0–5), class II (6–7), and class III (>7) respectively. Chest radiographs showed bilateral involvement, area of consolidation >50%, cavitation, effusion, and miliary nodules in 51.3%, 21.3%, 30.0%, 31.3%, and 3.8% respectively. Twenty-one (26%) patients developed respiratory failure requiring oxygen support, and 8 (10%) were admitted to ICU or died. The median (IQR) length-of-stay was 22 (13–36) days for the survivors. All patients were given anti-TB treatment.

### Cytokines/chemokines, HMGB1, RAGE, and inflammasome activation in PTB

As shown in Table 1, we found significantly higher plasma concentrations of IL-8/CXCL8 and IL-6 in patients with active PTB (~1.7–3.0 folds), when compared with IGRA-positive and IGRA-negative asymptomatic individuals. Plasma sTNFR1 and IL-18 concentrations were higher in the MTB infected individuals.

We found that transmembrane RAGE on dendritic cells and monocyte surfaces was increased, and RAGE and HMGB1 gene expressions were upregulated in active PTB (~1.2–2.8 fold); whereas the decoy, plasma soluble RAGE was significant depleted (Table 2). There was a trend of increase of plasma HMGB1 level in active PTB patients (~1.1 and 1.6 fold higher than IGRA-positive and IGRA-negative individuals respectively), but the comparisons did not reach statistical significance. The inflammasome-related Caspase-1 activity and NALP3 gene expression were shown to be increased. We found significant correlations between RAGE expression and plasma proinflammatory cytokine levels (IL-8/CXCL8, IL-6, sTNFR1; Table 2 footnotes).

### Bacteriological and clinical correlations

As shown in Table 3, we found significant correlations between mycobacterial load (as indicated by the semi-quantitative smear results and ‘time-to-culture positivity’) and plasma IL-8/CXCL8 and IL-6 concentrations in patients with active PTB. IL-8/CXCL8 showed strong correlations with extent of lung consolidation (*r*_s_ +0.509, P<0.001), duration of hospitalization (*r*_s_ +0.407, P<0.001), and TB severity scores; similar results were shown for IL-6. Significant associations were found between RAGE, HMGB1 and NALP3 gene expressions, and extent of consolidation (Table 3 footnotes). Plasma HMGB1 concentration correlated with fever duration (*r*_s_ +0.272, P = 0.015).

**Fig 1:**
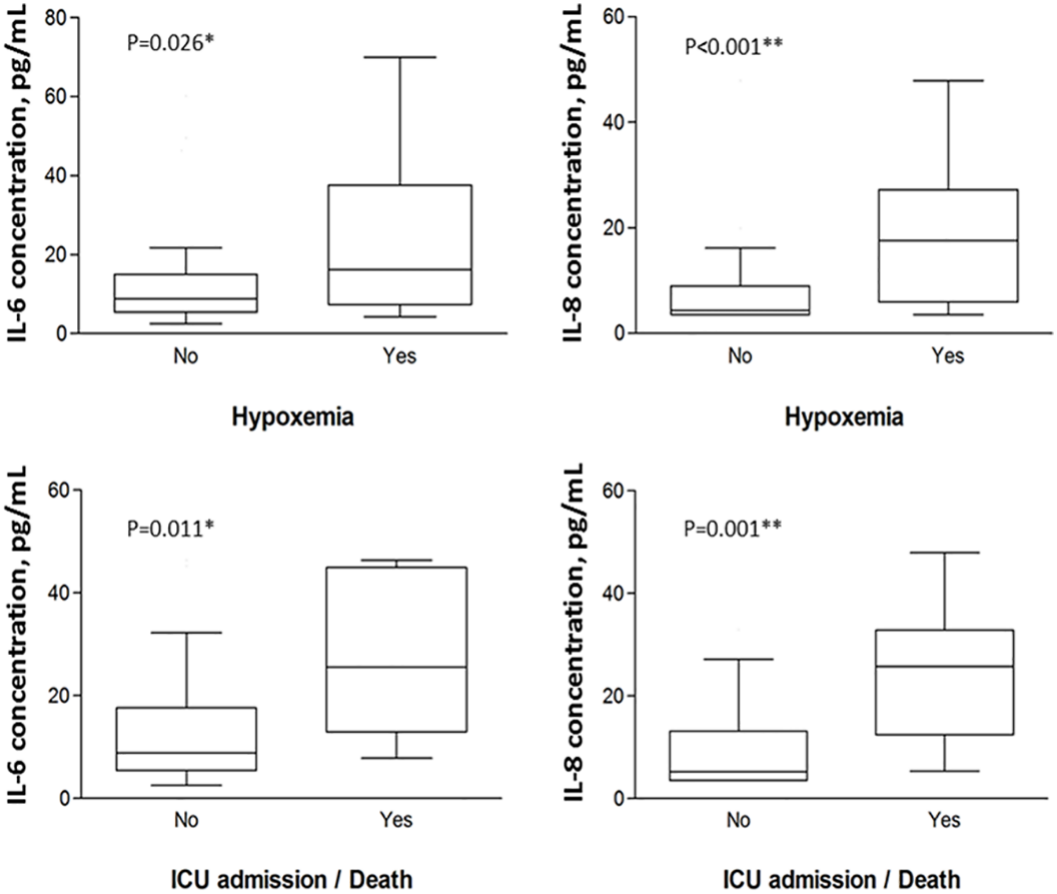
High plasma IL-6 and IL-8/CXCL8 concentrations were significantly associated with development of respiratory failure (hypoxemia, upper panels), and the adverse outcomes of ICU admission/death (lower panels) in patients with confirmed active PTB (n = 80). **Note**. Other cytokines, hypoxemia [yes *vs* no]: IL-18 [median (IQR), 1655.0 (1266.6–2185.8) *vs* 1516.3 (1285.8–1882.3) pg/mL]; sTNFR1 [median (IQR), 1460.2 (1054.1–2008.7) *vs* 1664.6 (1089.2–2571.4) pg/mL; HMGB1 [median(IQR), 3.5(2.1–5.2) *vs* 3.2 (1.7–4.5)], outliers excluded; all P >0.05.

As shown in Fig 1, high plasma IL-8/CXCL8 [median(IQR), 17.6(5.9–27.3) *vs* 4.3(3.6–9.0) pg/mL, P<0.001) and IL-6 [16.2(7.4–37.7) *vs* 8.7(5.5–15.0) pg/mL, P = 0.026] concentrations were significantly associated with development of respiratory failure; the same cytokines were also associated with ICU admission/death. Patients with respiratory failure showed trend of increase in HMGB1 [plasma concentration, median(IQR), 3.5(2.1–5.2) *vs* 3.2(1.7–4.5); 10^th^–90^th^ percentile, 1.6–13.5 *vs* 1.0–6.0 ng/mL; gene expression RQ, 1.4(0.9–3.1) *vs* 1.0(0.6–3.0)]. After adjustment for severity score at presentation, age, gender, and comorbidity, IL-8/CXCL8 (adjusted OR 1.12 per unit increase, 95%CI 1.02–1.23, P = 0.021) and HMGB1 (adjusted OR 1.42 per unit increase, 95%CI 1.08–1.87, P = 0.012) concentrations were shown to be independently associated with respiratory failure (Table 4a and 4b). A separate model accounting for the same confounders showed that IL-8/CXCL8 (adjusted OR 1.14 per unit increase, 95%CI 1.01–1.30, P = 0.041) and HMGB1 (adjusted OR 1.44 per unit increase, 95%CI 0.97–2.13, P = 0.074) independently predicted ICU admission/death.

**Table 4:** Independent variables associated with development of respiratory failure in active PTB, as shown in final multivariable logistic regression models.

**(a) Model 1: plasma HMGB1 concentration as a covariate**			
**Variable**	**Adjusted Odds Ratio (OR)**	**95% Confidence Interval (CI)**	**P-value**
**Age, per 20 years**	2.57	1.28–5.13	0.008
**Sex, male**	0.40	0.10–1.60	0.193
**Comorbidity** ^1^	2.41	0.63–9.20	0.198
**TB score** ^2^			
**class II**	9.32	1.97–44.2	0.005
**class III**	11.17	1.81–69.2	0.009
**HMGB1** ^3^	1.33	1.05–1.68	0.020
**(b) Model 2: plasma HMGB1 and cytokine/chemokine concentrations as covariates**			
**Variable**	**Adjusted Odds Ratio (OR)**	**95% Confidence Interval (CI)**	**P-value**
**Age, per 20 years**	2.82	1.26–6.29	0.011
**Sex, male**	0.15	0.03–0.84	0.032
**Comorbidity** ^1^	1.73	0.38–7.85	0.478
**TB score** ^2^			
**class II**	8.19	1.47–45.51	0.016
**class III**	4.00	0.47–33.94	0.204
**HMGB1** ^3^	1.42	1.08–1.87	0.012
**IL-6**	1.00	0.97–1.03	0.770
**IL-8/CXCL8** ^3^	1.12	1.02–1.23	0.021
**IL-18**	1.00	1.00–1.00	0.530

^**1**^ Comorbidity: presence of major comorbidities as defined in the Charlson’s comorbidity index;

^**2**^ TB severity score, constructed based on 13 symptoms and clinical signs (Class II score 6–7, Class III score 8–13; compared with Class I score 0–5);

^**3**^ HMGB1: per ng/mL increase, IL-8/CXCL8: per pg/mL increase [NB. including the variable ‘symptom duration prior to presentation’ as a covariate did not change the results.]

### Ex vivo cytokine response upon stimulation with recombinant-HMGB1 and MTB-antigen

**Fig 2:**
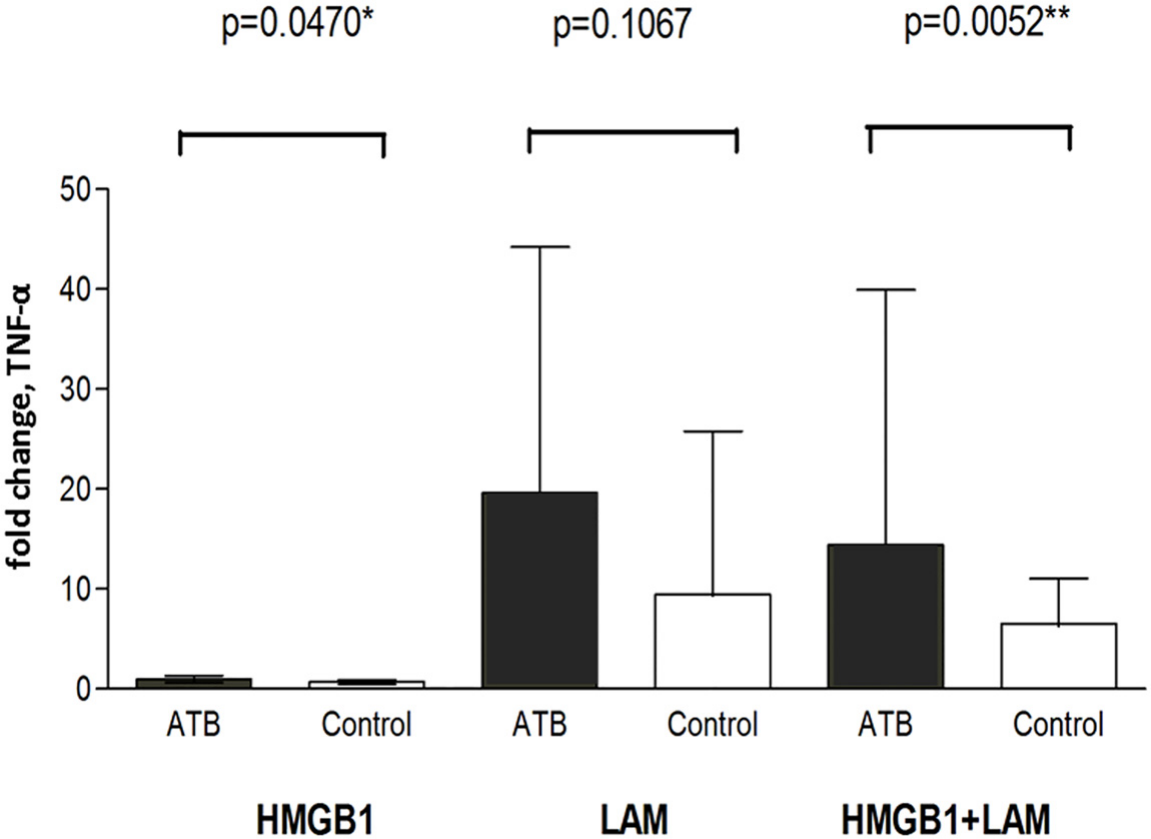
*Ex vivo* stimulation of PBMC with recombinant-HMGB1 alone, LAM alone and their combinations. TNF-α release was substantially higher with HMGB1 and LAM co-stimulation; the response in active PTB patients was about 2 times greater than in the uninfected (PTB *vs* IGRA-negative subjects, median(IQR) fold-change, 14.4(4.7–39.9) *vs* 6.6(2.6–11.1), P = 0.005). **Footnotes**: ATB, patients with active PTB; Control, IGRA-negative individuals. Fold change: TNF-α release with/without ligand stimulation. Box and whisker represent 50^th^ and 75^th^ percentiles respectively; Mann Whitney U test. Median fold-change of TNF-α with HMGB1+LAM stimulation in ATB cases *vs* all IGRA-positive/negative individuals: 14.4 (4.7–39.9) vs 6.1 (2.8–11.0), P = 0.001.

When stimulated with recombinant-HMGB1 alone, PBMC from active PTB patients showed a small but significantly greater response in releasing TNF002Dα compared with the uninfected individuals (PTB *vs* IGRA-negative subjects: fold-change >1.0, in 44.3% *vs* 22.2%, P = 0.042; *vs* IGRA-positive/negative subjects 28.9%, P = 0.090). When co-stimulated with MTB antigen (i.e., HMGB1 + LAM), a substantial increase in TNF-α release was observed in active PTB patients; and the response was about 2 times greater than the uninfected (PTB *vs* IGRA-negative subjects, median(IQR) fold-change, 14.4(4.7–39.9) *vs* 6.6(2.6–11.1), P = 0.005; PTB *vs* IGRA-positive/negative subjects, 14.4(4.7–39.9) *vs* 6.1(2.8–11.0), P = 0.001) (Fig 2). When stimulated with ODN (a TLR9-specific ligand), no significant cytokine stimulation was observed. Results for other cytokines (e.g. IL-18, IL-10) with HMGB1/LAM stimulation are provided in S2 File.

## Discussion

Our results showed increased pro-inflammatory cytokines and activation of the HMGB1/RAGE signaling pathway in active PTB, which correlated with clinico-/radiographical severity. HMGB1 may potentiate the inflammatory responses induced by MTB antigens. Such findings shed light on the pathogenesis of severe TB, and may assist hypothesis generation for the development of new management and control strategies.

We found significant elevation of the pro-inflammatory cytokines IL-8/CXCL8 and IL-6 in patients with active PTB; IL-18 and sTNFR-1 (indicating TNF-α release) were also increased in infected individuals. Notably, plasma levels of these proinflammatory cytokines (e.g. IL-8/CXCL8, IL-6) were shown to significantly correlate with mycobacterial load, extent of radiographic consolidation, TB severity score, and durations of fever and hospitalization. Further, multivariate analyses showed that IL-8/CXCL8 concentration was an independent predictor for development of respiratory failure (adjusted OR 1.12 per unit rise, 95%CI 1.02–1.23) adjusted for confounders such as old age and underlying comorbidities. Associations with adverse outcomes of ICU admission/death were also observed. Besides enhancing tissue injury, IL-8/CXCL8, as well as IL-6 and IL-18 have been shown in experimental MTB infections to promote macrophage and T-cell–mediated anti-mycobacterial immunity such as IFN-γ secretion and recruitment of inflammatory cells. TNF-α is known to be crucial in mycobacterial control including granuloma formation [5,31,32]. However few studies have shown clinical relevance despite their frequent detection in TB patients; in one report, high IL-8/CXCL8 concentration in bronchoalveolar lavage fluids and its association with fatality was described [31,33–37]. Our clinical data provide important support for the pathogenic roles of pro-inflammatory cytokines in active PTB, and suggest further evaluation of their clinical utility for prognostication and treatment response monitoring (as ‘biomarker’, e.g. IL-8/CXCL8) [35,37–42].

We found evidence of HMGB1/RAGE signaling pathway activation in active PTB. HMGB1 and RAGE genes were both upregulated; surface expression of transmembrane RAGE on the immune cells was increased [8,10,12,43]. Although only a slight increase in the circulating level of HMGB1 was detected (see section on Limitations), its decoy receptor soluble RAGE, was found to be significantly depleted [8,17,44]. Increased expression of RAGE correlated with plasma cytokine levels, and extent of lung consolidation (similar results for HMGB1). In multivariable analyses which controlled for clinical confounders and cytokine levels, HMGB1 was shown to be an independent predictor for severe outcomes (adjusted odds ratio ~1.4 per unit rise). In animal models of PTB, increased expressions of HMGB1 (and pro-inflammatory cytokines) were detected in the lungs, correlating with degree of tissue damage [18]. Its pathogenic role has been described recently in other infectious diseases including community-acquired pneumonia [16], influenza [45], skin and soft tissue [46] and intra-abdominal infections [17],contributing to serious complications or fatal outcomes. Being a receptor for HMGB1, signaling through RAGE activates the NF-κB pathway (possibly via ERK and p38-MAPK), which promotes transcription of the pro-inflammatory genes (e.g. IL-8/CXCL8, IL-6, TNF-α) in lung and immune cells [8,10,12,44]. Recent animal data suggest that RAGE may play a role in mycobacterial control [40]. Soluble RAGE is proposed to function as a counteractive, ligand-binding decoy [8,17,44],and depleted plasma level has been associated with excessive inflammation in respiratory diseases [47–49]. We first report inflammasome hyperactivation (NALP3 gene up-regulation and increased Caspase-1 activity) in active PTB patients. Experimental data suggest that secretion of HMGB1 and several inflammatory cytokines (e.g. IL-18) in severe sepsis is inflammasome-dependent [8,10,12,14]. Recently, MTB has been shown to specifically activate the NALP3-inflammasome to trigger the inflammation cascade in mice [50].

These observations are further supported by our *ex vivo* study results. A small but significant TNF-α response was detected in the PTB cases with recombinant-HMGB1 stimulation alone; however when combined with MTB antigen (LAM), a substantial response was shown (double that of the uninfected), indicating a possible additive/synergistic effect. Stimulation with another ligand, ODN, either alone or in combination with HMGB1, did not produce any significant effect (data not shown). HMGB1 is known to interact with various microbial antigens to increase its binding and pro-inflammatory actions [5,10,11,12]. Consistent with our findings, potentiation of cytokine induction (e.g. TNF-α) had been shown in experimental *M*. *bovis* BCG infection with the addition of HMGB1 [18].

Collectively, our results suggest that innate immune mechanisms play important pathogenic roles in active PTB, which warrant more detailed characterization [8,10,11,12,32]. Besides implications for disease monitoring [16,31], such data may provide basis for the on-going research on novel adjunctive therapies (e.g. anti-HMGB1 monocloncal antibodies, HMGB1-binding small molecules, RAGE-binding molecules as competitive antagonists, PPARγ activation to attenuate HMGB1 production) to reduce excessive inflammation in TB disease [5,7,8,10,11,17,51],and next-generation TB vaccines with enhanced immunogenicity (e.g. HMGB1-adjuvanted TB subunit vaccine) [9,32].

The strengths of this study include its prospective, case-control design; active TB, latent TB, and the uninfected were compared; events occurring along the proposed signaling pathway (inflammasome–HMGB1/RAGE–cytokines) were investigated at multiple levels (plasma, cell surface, gene expression) to provide a more comprehensive picture. However, additional studies are required to ascertain their causal relationships, as well as the other related cytokines/chemokines and receptors (e.g. IL-17, IFN-γ, CCL2/MCP-1, CXCL10/IP-10; the toll-like receptors) [5,8,10,27]. Unlike *in vitro* experiments, detection of circulating levels of certain cytokines (e.g. IL-1β, IL-10, TNF-α) can be challenging; and circulating ‘receptor-bound’ HMGB1 (e.g. bound to soluble RAGE) cannot be measured accurately with the current assays [16,20,25,26]. As such, gene expressions were also studied. Since HMGB1 can promote or suppress inflammation and the immune response depending on its redox state [52],further study to determine reduced/oxidized HMGB1 by Western blotting is also warranted. We could not study lung tissues as obtaining these specimens were infeasible in most TB patients. Follow up studies on HMGB1/RAGE targeting to attenuate cytokine responses, and serial monitoring of immunological parameters during the course of anti-TB treatment have been planned.

In conclusion, our results showed that in patients with active PTB, there was activation of HMGB1/RAGE signaling and increased pro-inflammatory cytokine responses which correlated with disease severity. Such findings has important implications on pathogenesis and provide basis for the development of new strategies for disease monitoring, management and control.

## Supporting Information

S1 File Assays of plasma HMGB1, soluble RAGE, and cytokines/chemokines concentrations. (PDF)

S2 File *Ex vivo* cytokine responses of PBMC upon stimulation with recombinant HMGB1 and LAM. (PDF)

## References

[1] World Health Organization. Global tuberculosis report 2015. http://www.who.int/tb/publications/global_report/en/. Accessed 20th May, 2016.

[2] Lawn SD, Zumla AI. Tuberculosis. Lancet 2011; 378: 57–72. 10.1016/S0140-6736(10)62173-3 21420161

[3] Hansel NN, Merriman B, Haponik EF, Diette GB. Hospitalizations for tuberculosis in the United States in 2000: predictors of in-hospital mortality. *Chest* 2004; 126: 1079–86. 15486367 10.1378/chest.126.4.1079

[4] Lui G, Wong RY, Li F, Lee MKP, Lai RWM, Li TCM, et al High mortality in adults hospitalized for active tuberculosis in a low HIV prevalence setting. *PLoS One* 2014; 9: e92077 10.1371/journal.pone.0092077 24642794 PMC3958438

[5] Dheda K, Schwander SK, Zhu B, van Zyl-Smit RN, Zhang Y. The immunology of tuberculosis: from bench to bedside. *Respirology* 2010; 15: 433–50. 10.1111/j.1440-1843.2010.01739.x 20415982 PMC5463744

[6] Verrall AJ, Netea MG, Alisjahbana B, Hill PC, van Crevel R. Early clearance of Mycobacterium tuberculosis: a new frontier in prevention. *Immunology* 2014; 141: 506–13. 24754048 10.1111/imm.12223 PMC3956425

[7] Musumeci D, Roviello GN, Montesarchio D. An overview on HMGB1 inhibitors as potential therapeutic agents in HMGB1-related pathologies. *Pharmacol*. *Ther*. 2014; 141: 347–357. 10.1016/j.pharmthera.2013.11.001 24220159

[8] Lotze MT, Tracey KJ. High-mobility group box 1 protein (HMGB1): nuclear weapon in the immune arsenal. *Nat*. *Rev*. *Immunol*. 2005; 5: 331–42. 15803152 10.1038/nri1594

[9] Grover A, Troudt J, Foster C, Basaraba R, Izzo A. High mobility group box 1 acts as an adjuvant for tuberculosis subunit vaccines. *Immunology* 2014; 142: 111–23. 10.1111/imm.12236 24350616 PMC3992053

[10] Sims GP, Rowe DC, Rietdijk ST, Herbst R, Coyle AJ. HMGB1 and RAGE in inflammation and cancer. *Annu*. *Rev*. *Immunol*. 2010; 28: 367–88. 10.1146/annurev.immunol.021908.132603 20192808

[11] Castiglioni A, Canti V, Rovere-Querini P, Manfredi AA. High-mobility group box 1 (HMGB1) as a master regulator of innate immunity. *Cell Tissue Res*. 2011; 343: 189–99. 10.1007/s00441-010-1033-1 20835834

[12] Keyel PA. How is inflammation initiated? Individual influences of IL-1, IL-18 and HMGB1. *Cytokine* 2014; 69: 136–145. 10.1016/j.cyto.2014.03.007 24746243

[13] Willingham SB, Allen IC, Bergstralh DT, Brickey WJ, Huang MT, Taxman DJ, et al NALP3 facilitates in vivo caspase-1 activation, necrosis, and HMGB1 release via inflammasome-dependent and -independent pathways. *J*. *Immunol*. 2009; 183: 2008–15. 10.4049/jimmunol.0900138 19587006 PMC3652593

[14] Lamkanfi M, Sarkar A, Vande Walle L, Vitari AC, Amer AO, Wewers MD, et al Inflammasome-dependent release of the alarmin HMGB1 in endotoxemia. *J*. *Immunol*. 2010; 185: 4385–92. 10.4049/jimmunol.1000803 20802146 PMC3428148

[15] Tian J, Avalos AM, Mao SY, Chen B, Senthil K, Wu H, et al Toll-like receptor 9-dependent activation by DNA-containing immune complexes is mediated by HMGB1 and RAGE. *Nat*. *Immunol*. 2007; 8: 487–96. 17417641 10.1038/ni1457

[16] Angus DC, Yang L, Kong L, Kellum JA, Delude RL, Tracey KJ, et al Circulating high-mobility group box 1 (HMGB1) concentrations are elevated in both uncomplicated pneumonia and pneumonia with severe sepsis. *Crit*. *Care*. *Med*. 2007; 35: 1061–7. 17334246 10.1097/01.CCM.0000259534.68873.2A

[17] Kocsis AK, Szabolcs A, Hofner P, Takács T, Farkas G, Boda K, et al Plasma concentrations of high-mobility group box protein 1, soluble receptor for advanced glycation end-products and circulating DNA in patients with acute pancreatitis. *Pancreatology* 2009; 9: 383–91. 10.1159/000181172 19451748

[18] Grover A, Taylor J, Troudt J, Keyser A, Sommersted K, Schenkel A, et al Mycobacterial infection induces the secretion of high-mobility group box 1 protein. *Cell Microbiol*. 2008; 10: 1390–404. 10.1111/j.1462-5822.2008.01135.x 18331466

[19] Hofner P, Seprényi G, Miczák A, Buzás K, Gyulai Z, Medzihradszky KF, et al High mobility group box 1 protein induction by Mycobacterium bovis BCG. *Mediators Inflamm*. 2007; 2007: 53805 10.1155/2007/53805 18288272 PMC2233895

[20] Magryś A, Paluch-Oleś J, Kozioł-Montewka M, Zaborowski T, Milanowski J, Maciejewska B. Evaluation of high-mobility group box 1 protein concentration in serum of patients with M. tuberculosis infection. *Immunol*. *Invest*. 2013; 42: 49–60. 10.3109/08820139.2012.723769 23231044

[21] Lui G, Lee N, Cheung SW, Lam JS, Wong BC, Choi KW, et al Interferon gamma release assay for differentiating tuberculosis among pneumonia cases in acute healthcare setting. *J*. *Infect*. 2011; 62: 440–7. 10.1016/j.jinf.2011.04.011 21575991

[22] Tuberculosis and Chest Services, Centre for Health Protection, Hong Kong SAR. Tuberculosis Manual 2006. http://www.info.gov.hk/tb_chest/doc/Tuberculosis_Manual2006.pdf. Accessed 10 March 2011.

[23] Charlson ME, Pompei P, Ales KL, MacKenzie CR. A new method of classifying prognostic comorbidity in longitudinal studies: development and validation. *J*. *Chronic Dis*. 1987; 40: 373–83. 3558716 10.1016/0021-9681(87)90171-8

[24] Wejse C, Gomes VF, Rabna P, Gustafson P, Aaby P, Lisse IM, et al Vitamin D as supplementary treatment for tuberculosis: a double-blind, randomized, placebo-controlled trial. *Am*. *J*. *Respir*. *Crit*. *Care Med*. 2009; 179: 843–50. 10.1164/rccm.200804-567OC 19179490

[25] Lee N, Wong CK, Chan PKS, Chan MC, Wong RY, Lun SW, et al Cytokine Response Patterns in Severe Pandemic 2009 H1N1 and Seasonal Influenza among Hospitalized Adults. *PLoS One* 2011; 6: e26050 10.1371/journal.pone.0026050 22022504 PMC3192778

[26] Lee N, Wong CK, Chan PKS, Lun SW, Lui G, Wong B, et al Hypercytokinemia and Hyperactivation of phospho-p38 Mitogen-Activated Protein Kinase in Severe Human Influenza A Virus Infections. *Clin*. *Infect*. *Dis*. 2007; 45: 723–731. 17712756 10.1086/520981

[27] Lee N, Wong CK, Hui DSC, Lee SK, Wong RY, Ngai KL, et al Role of Human Toll-like Receptors in Naturally Occurring Influenza A Infections. *Influenza and Other Resp*. *Vir*. 2013; 7: 666–75. 10.1111/irv.12109 PMC5781199 23552014

[28] Lee N, Chan PK, Wong CK, Wong KT, Choi KW, Joynt GM, et al Viral clearance and inflammatory response patterns in adults hospitalized for pandemic 2009 influenza A(H1N1) virus pneumonia. *Antivir*. *Ther*. 2011; 16:237–47. 10.3851/IMP1722 21447873

[29] Ralph AP, Ardian M, Wiguna A, Maguire GP, Becker NG, Drogumuller G, et al A simple, solid, numerical score for grading chest x-ray severity in and out smear positive pulmonary tuberculosis. *Thorax* 2010; 65:863–869. 10.1136/thx.2010.136242 20861290

[30] Palaci M, Dietze R, Hadad DJ, Ribeiro FK, Peres RL, Vinhas SA, et al Cavitary disease and quantitative sputum bacillary load in cases of pulmonary tuberculosis. *J*. *Clin*. *Microbiol*. 2007; 45: 4064–6. 17928422 10.1128/JCM.01780-07 PMC2168542

[31] Azad AK, Sadee W, Schlesinger LS. Innate immune gene polymorphisms in tuberculosis. *Infect*. *Immun*. 2012; 80: 3343–59. 10.1128/IAI.00443-12 22825450 PMC3457569

[32] Kaufmann SH. How can immunology contribute to the control of tuberculosis? *Nat*. *Rev*. *Immunol*. 2001; 1: 20–30. 11905811 10.1038/35095558

[33] Ma X, Reich RA, Wright JA, Tooker HR, Teeter LD, Musser JM, et al Association between interleukin-8 gene alleles and human susceptibility to tuberculosis disease. *J*. *Infect*. *Dis*. 2003; 188: 349–55. 12870115 10.1086/376559

[34] Kassa D, Ran L, Geberemeskel W, Tebeje M, Alemu A, Selase A, et al Analysis of immune responses against a wide range of Mycobacterium tuberculosis antigens in patients with active pulmonary tuberculosis. *Clin*. *Vaccine Immunol*. 2012; 19: 1907–15. 10.1128/CVI.00482-12 23015647 PMC3535869

[35] Masood KI, Rottenberg ME, Salahuddin N, Irfan M, Rao N, Carow B, et al Expression of M. tuberculosis-induced suppressor of cytokine signaling (SOCS) 1, SOCS3, FoxP3 and secretion of IL-6 associates with differing clinical severity of tuberculosis. *BMC Infect*. *Dis*. 2013; 13: 13 10.1186/1471-2334-13-13 23320781 PMC3562147

[36] Santucci N, D'Attilio L, Kovalevski L, Bozza V, Besedovsky H, del Rey A, et al A multifaceted analysis of immune-endocrine-metabolic alterations in patients with pulmonary tuberculosis. *PLoS One* 2011; 6: e26363 10.1371/journal.pone.0026363 22022605 PMC3192801

[37] Hasan Z, Cliff JM, Dockrell HM, Jamil B, Irfan M, Ashraf M, et al CCL2 responses to Mycobacterium tuberculosis are associated with disease severity in tuberculosis. *PLoS One* 2009; 4: e8459 10.1371/journal.pone.0008459 20041183 PMC2793516

[38] Alessandri AL, Souza AL, Oliveira SC, Macedo GC, Teixeira MM, Teixeira AL. Concentrations of CXCL8, CXCL9 and sTNFR1 in plasma of patients with pulmonary tuberculosis undergoing treatment. *Inflamm*. *Res*. 2006; 55: 528–33. 17039284 10.1007/s00011-006-5136-9

[39] Ganachari M, Guio H, Zhao N, Flores-Villanueva PO. Host gene-encoded severe lung TB: from genes to the potential pathways. *Genes*. *Immun*. 2012; 13: 605–20. 10.1038/gene.2012.39 22992722 PMC3518758

[40] Chen T, Lin J, Wang W, Fleming J, Chen L, Wang Y, et al Cytokine and Antibody Based Diagnostic Algorithms for Sputum Culture-Positive Pulmonary Tuberculosis. *PLoS One*. 2015;10(12):e0144705 10.1371/journal.pone.0144705 26674517 PMC4682626

[41] Abhimanyu, Bose M, Varma-Basil M, Jain A, Sethi T, Tiwari PK, et al Establishment of Elevated Serum Levels of IL-10, IL-8 and TNF-β as Potential Peripheral Blood Biomarkers in Tubercular Lymphadenitis: A Prospective Observational Cohort Study. *PLoS One*. 2016;11(1):e0145576 10.1371/journal.pone.0145576 26784906 PMC4718686

[42] Krupa A, Fol M, Dziadek BR, Kepka E, Wojciechowska D, Brzostek A, et al Binding of CXCL8/IL-8 to Mycobacterium tuberculosis Modulates the Innate Immune Response. *Mediators Inflamm*. 2015;2015:124762 10.1155/2015/124762 26300588 PMC4537748

[43] van Zoelen MA, Wieland CW, van der Windt GJ, Florquin S, Nawroth PP, Bierhaus A, et al Receptor for advanced glycation end products is protective during murine tuberculosis. *Mol*. *Immunol*. 2012; 52: 183–9. 10.1016/j.molimm.2012.05.014 22698798

[44] Yang D, Chen Q, Yang H, Tracey KJ, Bustin M, Oppenheim JJ. High mobility group box-1 protein induces the migration and activation of human dendritic cells and acts as an alarmin. *J*. *Leukoc*. *Biol*. 2007; 81: 59–66. 16966386 10.1189/jlb.0306180

[45] Momonaka H, Hasegawa S, Matsushige T, Inoue H, Kajimoto M, Okada S, et al High mobility group box 1 in patients with 2009 pandemic H1N1 influenza-associated encephalopathy. *Brain Dev*. 2014; 36: 484–8. 10.1016/j.braindev.2013.07.001 23907181

[46] Johansson L, Snäll J, Sendi P, Linnér A, Thulin P, Linder A, et al HMGB1 in severe soft tissue infections caused by Streptococcus pyogenes. *Front*. *Cell Infect*. *Microbiol*. 2014; 4: 4 10.3389/fcimb.2014.00004 24524027 PMC3906589

[47] Santilli F, Vazzana N, Bucciarelli LG, Davi G. Soluble forms of RAGE in human diseases: clinical and therapeutical implications. *Curr*. *Med*. *Chem*. 2009; 16: 940–952 19275604 10.2174/092986709787581888

[48] Sukkar MB, Wood LG, Tooze M, Simpson JL, McDonald VM, Gibson PG, et al Soluble RAGE is deficient in neutrophilic asthma and COPD. *Eur*. *Respir*. *J*. 2012; 39: 721–9. 10.1183/09031936.00022011 21920897

[49] Iwamoto H, Gao J, Pulkkinen V, Toljamo T, Nieminen P, Mazur W. Soluble receptor for advanced glycation end-products and progression of airway disease. *BMC Pulm*. *Med*. 2014; 14: 68 10.1186/1471-2466-14-68 24758342 PMC4021457

[50] Dorhoi A, Nouailles G, Jörg S, Hagens K, Heinemann E, Pradl L, et al Activation of the NLRP3 inflammasome by Mycobacterium tuberculosis is uncoupled from susceptibility to active tuberculosis. *Eur*. *J*. *Immunol*. 2012; 42: 374–84. 10.1002/eji.201141548 22101787

[51] Wang G, Liu L, Zhang Y, Han D, Liu J, Xu J, et al Activation of PPARγ attenuates LPS-induced acute lung injury by inhibition of HMGB1-RAGE levels. *Eur*. *J*. *Pharmacol*. 2014; 726: 27–32. 24485891 10.1016/j.ejphar.2014.01.030

[52] Hernández-Pando R, Barrios-Payán J, Mata-Espinosa D, Marquina-Castillo B, Hernández-Ramírez D, Bottasso OA, et al The Role of High Mobility Group Box 1 Protein (HMGB1) in the Immunopathology of Experimental Pulmonary Tuberculosis. *PLoS One* 2015; 10: e0133200 10.1371/journal.pone.0133200 26201072 PMC4511675

