# Supplementary material for: HMGB1/RAGE Signaling and Pro-Inflammatory Cytokine Responses in Non-HIV Adults with Active Pulmonary Tuberculosis
Source: PLoS One. 2016 Jul 19;11(7):e0159132. doi: 10.1371/journal.pone.0159132 (PMC4951129; doi:10.1371/journal.pone.0159132)
Supplement: S1 File — (PDF) [file pone.0159132.s001.pdf]

SUPPLEMENTARY MATERIAL-1

Assays of plasma HMGB1, soluble RAGE, and cytokines/chemokines concentrations. Plasma concentrations of interleukin(IL)-1β, IL-6, IL-8/CXCL8, IL-10, IL-12p70, and tumour necrosis factor(TNF)-α were assayed using cytometric bead array (CBA) reagents (BD Pharmingen Corp., San Diego, CA, USA) with four-color FACSCalibur flow-cytometer (BD Biosciences Corp, San Jose, CA, USA), as previously described [25-27]. Plasma concentrations of HMGB1, soluble RAGE (soluble receptor of HMGB1), IL-18, and sTNFR-1 (soluble tumour necrosis factor receptor-1, which indirectly indicating TNF-α release) were measured using enzyme-linked immunosorbent assays (Shino-Test, Kanagawa, Japan; R&D systems, Minneapolis, MN, USA; and MBL International Corp., Des Plaines, IL, USA). These cytokines/chemokines were selected for study based on their reported significance in active PTB [5,6,31].

Flow cytometric assay of transmembrane RAGE on monocytes and dendritic cells surface. Expression of transmembrane RAGE on surface of peripheral blood monocytes (CD14+), myeloid dendritic cells/mDC (CD16-CD14-ILT3+CD33+) and plasmacytoid dendritic cells/pDC (CD16-CD14-ILT3+CD33-) was assessed by flow cytometric analysis using established methods [27]. Unconjugated polyclonal rabbit anti-human RAGE antibody for flow analysis (Millipore Co., MA, USA), and PE-conjugated goat anti-rabbit IgG (H+L) secondary antibody (Invitrogen Corp., CA, USA) were used for the detection of RAGE. The CD14+ gated population was set to identify the monocyte population. For the analysis of DC, FITC-conjugated CD14 and CD16, PC7-conjugated immunoglobulin-like transcript (ILT)-3/CD85k/CD1c/BDCA2 (Beckman Coulter Inc, CA, USA), and APC-conjugated CD33 (BD Biosciences, San Jose, CA, USA) were used. The DC population was identified as CD14 and CD16 double negative and ILT3 positive population; mDCs and pDCs were differentiated by high and low CD33 expression, respectively. To obtain 10,000 DCs, 200,000 of monocytes were gated for analysis with 4-color FACSCalibur flow cytometer (BD Biosciences Corp, San Jose, CA, USA). All results were expressed in Mean Fluorescence Intensity (MFI).

Figure S1. Representative dot plots for the flow cytometry analysis of the RAGE expression on different subtypes of DCs. (**a**) Representative dot plots showing the gating of CD14-CD16-cells within the monocyte population. (**b-d**) Representative dot plots showing the expression of RAGE on CD14-CD16-ILT3+ total DCs, CD14-CD16-ILT3+CD33+ mDCs, and CD14-CD16-ILT3+CD33- pDCs, respectively.

(a)

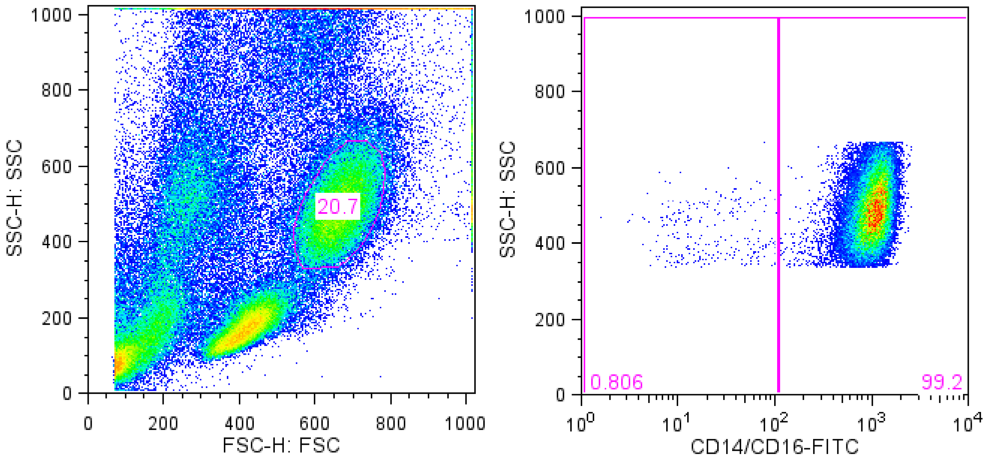

(b)

CD14/CD16-ILT3+ **Total DCs**

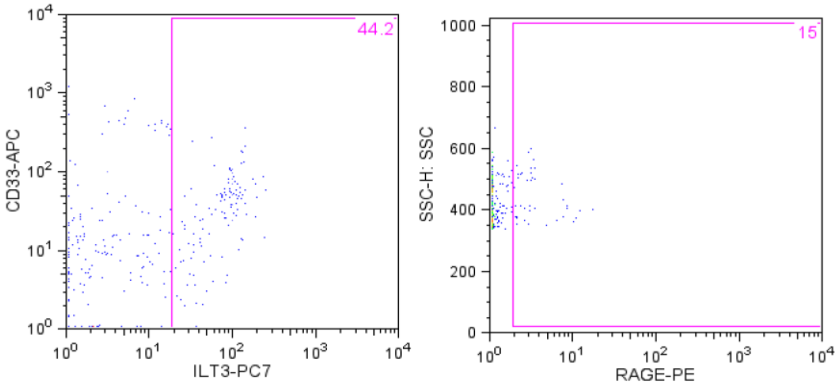

(c)

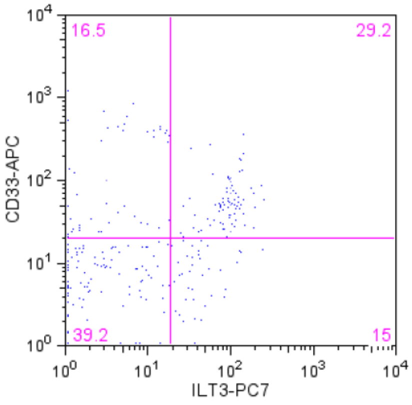

CD14/CD16-ILT3+CD33+

mDCs

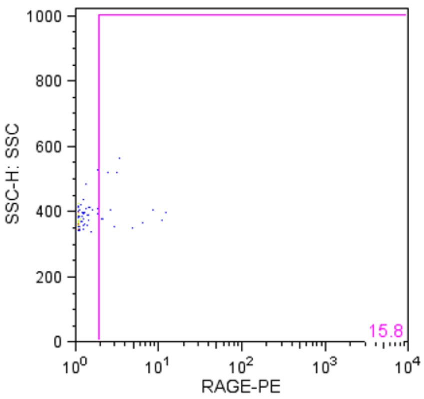

(d)

CD14/CD16-ILT3+CD33-

pDCs

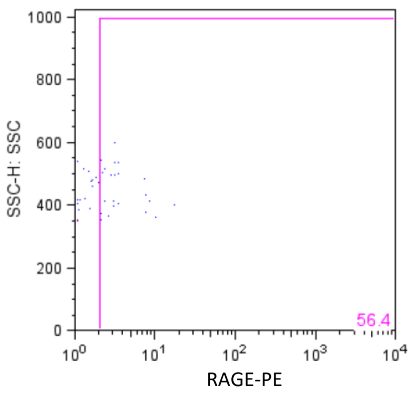

RAGE-PE

Real-time quantitative PCR assay of mRNA expression of HMGB1, RAGE, and NALP3 genes. Total RNA of PBMC was extracted by RNeasy Mini Kit (Qiagen Inc., Canada). After pre-treatment with deoxyribonuclease I (Invitrogen Corp., CA, USA), RNA samples were stored at –70°C for batch analysis. For each reaction, approximately 0.5 μg of total RNA was reversely transcribed to complementary DNA (cDNA) with reverse transcription reagents (Applied Biosystems). The mRNA expression of HMGB1, RAGE, and the inflammasome-related NALP3 genes was measured by real-time RT-PCR assay using the Applied Biosystems 48-well StepOne™ Real Time PCR System (Applied Biosystems Inc., CA, USA), containing 30 μM of HMGB1 5' and 3' primers (5'-TTTCAAACAAAGATGCCACA-3', 5'-GTTCCCTAAACTCCTAAGCAGATA-3'), or RAGE 5' and 3' primers (5'-ACAGCAGTTGGAGCCTGGGTG-3', 5'-AAGAGGGAGCCGTTGGGAAG-3'), or NALP3 5' and 3' primers (5'-CTTCTCTGATGAGGCCCAAG-3', 5'-GCAGCAAACTGGAAAGGAAG-3') respectively; and GADPH 5' and 3' primers (5'- ATGGGGAAGGTGAAGGTCG-3, 5'-GGGGTCATTGATGGCAACAATA-3'), 1 x FastStart Universal SYBR Green Master (ROX) (Roche Applied Science, Mannheim, Germany), and cDNA template. The relative gene expression of HMGB1, RAGE, and NALP3 was calculated by comparing with the housekeeping GAPDH gene, and expressed as Relative Quantitation (RQ) values [26,27]. In addition, assay of Caspase-1 (inflammasome-related) activity in PBMC was performed using Caspase-1 colorimetric assay kit (Millipore Corp., MA, USA).

Ex vivo stimulation studies with recombinant-HMGB1 and Lipoarabinomannan (LAM). Human PBMC were isolated using a density gradient centrifugation (Ficoll-Paque Plus; GE Healthcare Life Sciences, NJ, USA). PBMC (5×10^6/well) were distributed into 96-well plates (Corning Inc. Costar, NY, USA), and incubated in a 5% CO_2 incubator at 37°C in 1 ml of RPMI-1640 medium, supplemented with 10 % (v/v) fetal calf serum (FCS) for 24 hours, with or without the following ligands: (1) recombinant-HMGB1 (R&D Systems); (2) lipoarabinomannans (LAM, a MTB cell wall related glycolipid, a TLR2 ligand; Invivogen Corp., CA, USA); (3) a combination of recombinant-HMGB1 and LAM (for any additive effect); and (4) oligodeoxynucleotide (CpG-ODNs, a TLR-9 ligand for comparison; Invivogen Corp., CA, USA), to assess for their effects on cytokine stimulation. Concentrations of cytokines/chemokines released into the culture supernatant were quantitated by CBA or ELISA as described above. The fold-change in cytokine release with/without ligand stimulation was calculated for individual cytokine (e.g. TNF-α concentration after incubation with

recombinant-HMGB1 / with medium alone). A value of >1.0 was considered as a positive response to ligand stimulation [25,27].
