# Supplementary material for: HMGB1/RAGE Signaling and Pro-Inflammatory Cytokine Responses in Non-HIV Adults with Active Pulmonary Tuberculosis
Source: PLoS One. 2016 Jul 19;11(7):e0159132. doi: 10.1371/journal.pone.0159132 (PMC4951129; doi:10.1371/journal.pone.0159132)
Supplement: S2 File — (PDF) [file pone.0159132.s002.pdf]

SUPPLEMENTARY MATERIAL-2

***Ex vivo* cytokine responses of PBMC upon stimulation with recombinant HMGB1 and LAM**

(1) HMGB1 alone, active PTB patients *vs* asymptomatic individuals
[results are expressed as percentage of subjects showing a positive response, ie, fold-change >1.0]:

TNF-α, 44.3% *vs* 28.9% (P=0.090)

IL-18, 57.0% *vs* 33.3% (P=0.011)*

IL-10, 11.4% *vs* 8.9% (P>0.05)

IL-6, 48.1% *vs* 53.3% (P>0.05)

IL-8/CXCL8, 22.8% *vs* 26.7% (P>0.05)

(2) HMGB1+LAM, active PTB patients *vs* asymptomatic individuals
[results are expressed as fold-change in cytokine release, median(IQR)]:

TNF-α, 14.4 (4.7-39.9) *vs* 6.1 (2.8-11.0) pg/mL (P=0.001)*

IL-18, 1.0 (1.0-1.2) *vs* 1.0 (1.1-1.3) pg/mL (P>0.05)

IL-10, 1.0 (1.0-3.7) *vs* 1.0 (1.0-1.0) pg/mL (P=0.006)*

IL-6, 102.2 (22.4-261.0) *vs* 58.3 (24.1-150.3) pg/mL (P>0.05)

IL-8/CXCL8, 1.0 (1.0-1.0) *vs* 1.0 (1.0-1.8) pg/mL; 95th percentile 518.0 vs 12.2 pg/mL (P>0.05)

[The magnitude of fold-change was compared as most subjects showed a cytokine concentration ratio >1.0, e.g. TNF-α, IL-6]
